# Defect formation in multiwalled carbon nanotubes under low-energy He and Ne ion irradiation

**DOI:** 10.3762/bjnano.9.186

**Published:** 2018-07-09

**Authors:** Santhana Eswara, Jean-Nicolas Audinot, Brahime El Adib, Maël Guennou, Tom Wirtz, Patrick Philipp

**Affiliations:** 1 Advanced Instrumentation for Ion Nano-Analytics (AINA), MRT Department, Luxembourg Institute of Science and Technology (LIST), 41 rue du Brill, L-4422 Belvaux, Luxembourg; 2 Materials Research and Technology Department, Luxembourg Institute of Science and Technology, 41 rue du Brill, L-4422 Belvaux, Luxembourg

**Keywords:** carbon nanotubes, helium ion microscope, ion irradiation, Raman, simulations

## Abstract

The mechanical, structural, electronic and magnetic properties of carbon nanotubes can be modified by electron or ion irradiation. In this work we used 25 keV He^+^ and Ne^+^ ion irradiation to study the influence of fluence and sample thickness on the irradiation-induced damage of multiwalled carbon nanotubes (MWCNTs). The irradiated areas have been characterised by correlative Raman spectroscopy and TEM imaging. In order to preclude the Raman contribution coming from the amorphous carbon support of typical TEM grids, a new methodology involving Raman inactive Au TEM grids was developed. The experimental results have been compared to SDTRIMSP simulations. Due to the small thickness of the MWCNTs, sputtering has been observed for the top and bottom side of the samples. Depending on thickness and ion species, the sputter yield is significantly higher for the bottom than the top side. For He^+^ and Ne^+^ irradiation, damage formation evolves differently, with a change in the trend of the ratio of D to G peak in the Raman spectra being observed for He^+^ but not for Ne^+^. This can be attributed to differences in stopping power and sputter behaviour.

## Introduction

Carbon nanotubes (CNTs) have been investigated intensively due to their excellent properties [1]. Modifying and tuning them by electron or ion irradiation is part of these studies [2]. Irradiation-induced defects affect the elastic modulus and the tensile strength of CNTs [3]. For example, for multiwalled carbon nanotubes (MWCNTs), the presence of a small number of defects can increase the interlayer shear strength by several orders of magnitude [4]. In general, single-wall carbon nanotubes (SWCNTs) have the tendency to group in bundles. By electron irradiation the different CNTs can be linked by inter-tube bridging, which allows the bending modulus to be increased by a factor 30 [5]. Similar results can be obtained by ion irradiation. Si^+^ irradiation was used to weld MWCNTs with the objective to create electronic connections between different tubes for CNT junctions [6]. Similarly, He^+^ irradiation has been used to interconnect double-wall carbon nanotubes (DWCNTs) by cross-linking in order to form a multiscale-structured composite with improved mechanical properties which is similar to the one of collagen found in nature [7]. Vacancy formation and interconnections forming between CNTs have also been observed in molecular dynamics (MD) simulations during the irradiation of SWCNTs supported by silica [8]. MD simulations have been used to show that ion irradiation with incidence angles closer to the surface normal favours the formation of double and multiple vacancies and in-plane disorder while more grazing incidence leads mainly to single vacancies and substitution, the latter being limited to chemical species reacting with carbon [9]. For He^+^ irradiation of graphene, Stone–Thrower–Wales defects form preferentially [10]. For graphene, 1000 eV proved also to be the optimum energy for defect and nanopore formation at an incidence angle of 60° with respect to the surface normal and of 400–500 eV for angles closer to the surface normal [11]. Besides modification of the mechanical properties, ion irradiation can also be used to change the shape of CNTs: suspended SWCNTs get straightened under ion irradiation [12]. Similarly, for a sheet of several layers of graphene suspended on a TEM grid, the initial roughness disappears under 30 keV He^+^ irradiation for a relatively low fluence of 8 × 10^14^ ions/cm^2^ before the sample turns amorphous [13]. Transforming graphene into fullerenes has been carried out by electron irradiation [14], and graphitic nanostripes have been obtained from SiC by MeV Ta or Pb irradiation [15]. In addition, ion irradiation can also be used to modify the electronic properties. The presence of defects can increase the resistivity by several orders of magnitude [2,16]. When combining low-fluence ion irradiation with subsequent annealing, the electrical conductivity of SWCNTs can be improved [17]. The appearance of magnetism was reported for graphite after proton irradiation [18] and of fullerenes after the irradiation with heavy ions [19].

For the development of novel technological applications, being able to modify the structure of CNTs alone is not sufficient. It is also important to relate the structural changes to the mechanical, electrical and magnetic properties. This is only possible when the structural changes can be well characterised. TEM imaging has been able to provide information on the structural damage as a function of fluence for a range of experimental conditions, including He^+^ [20–21], C^+^ [21–22], N^+^ [22], Si^+^ [22], Kr^+^ [23], and Ar^+^ irradiation [21–22]. The same is true for defects produced by electron irradiation [24]. Electron diffraction pattern can be used to quantify the degree of amorphisation [13]. Raman spectroscopy has also been widely used to investigate different carbon-based materials. It has been used to study the influence of the twist angle in combination with defects of bi-layered graphene on the Raman peaks [25], to correlate ion irradiation to the number of defects and the change in elastic modulus [7], the comparison of C^+^ and Ni^+^ ion irradiation for defect production in SWCNTs [26], the characterisation of composite materials containing MWCNTs [27], the implantation of Si and C ions into DWCNTs [28], and to differentiate between carbon materials with different sp^2^ environment [29]. The latter study focused on graphene, highly oriented pyrolytic graphite (HOPG) and SWCNT and the modification of the Raman spectra when introducing defects in the different materials. Furthermore, HOPG and SWCNT spectra differ from those of graphite and nanocarbon [1]. The Raman spectrum of MWCNTs is in between those of graphite and nanocarbon. All these materials have a D band peak around 1350 cm^−1^, a G band peak around 1590 cm^−1^ and G’ band peak in the region of 2700 cm^−1^. The first is related to the presence of disordered carbon, the second to the tangential vibrations of graphitic carbon and the third one to two-phonon scattering related to long-range order in the sample. Depending on the sample a fourth peak due to intercalated graphite compounds and increasing disorder produced by functionalization and strain can appear in the region of 1617–1625 cm^−1^ [1]. Further information on defects can be obtained by the intensity of optical absorbance which is directly related to the number of defects in CNTs [30], or X-ray photoelectron spectroscopy (XPS) which provides some information on the chemical environment of the carbon atoms [31].

In this context, it is to be noted that helium ion microscopy (HIM) has received increasing attention recently as a high-resolution imaging tool [32–33]. A He^+^ or Ne^+^ ion beam can be used to irradiated the samples with an impact energy in the range of 5 to 30 keV, either for imaging or nano-machining [34–35], or for doing both simultaneously [33]. For instance, the HIM has already been used for the imaging of graphene flakes [36] and their etching [37], as well as the imaging of SWCNTs [38]. After the development of a compact mass spectrometer for this instrument [39–40], the detection and imaging of sputtered ions can also be used for process control [41], including carbon-containing materials [42–43].

Whereas in previous studies, the modification of CNTs has been carried out using various dedicated experimental setups for ion-irradiation, HIM emerges as specially well suited for targeted modification and visualisation of such materials. Therefore, the goal of the present work is to investigate the structural modifications of MWCNTs by low energy He^+^ and Ne^+^ ion irradiation for fluences of 10^14^ to 10^18^ ions/cm^2^, i.e., for imaging conditions found on the HIM [44–46]. We present a correlative approach in which ion-irradiation-induced modifications are characterised by Raman spectroscopy and TEM imaging, and the experimental results are compared to numerical simulations to explain the different observations and to discuss the irradiation of suspended vs deposited MWCNTs and the influence of the thickness of a layer of suspended MWCNTs on the modifications. This correlative approach provides insights related to optimal imaging conditions for HIM as well as controlled and targeted structural modification of carbon using ion-irradiation. Numerical simulations will rely on the SDTRIMSP code [47], which is relying on the binary collision approximation (BCA). This decision is based on SDTRIMSP’s ability to model ion fluences found in experiments. Molecular dynamics (MD) simulations would be able to provide an atomistic description of the sputter processes, which has already been done for several systems [48], however experimental fluences are beyond their possibilities. BCA-based simulations have already been used for the sputtering of targets with a cylindrical geometry [49], however, as we are going to irradiate a slab of MWCNTs we prefer to use a solid slab representing the whole sample. A study by Hobler et al. showed that BCA-based simulations can be used to study backward and forward sputtering of membranes or targets with similar geometry as long as sample thickness not lower than 10–30% of the mean depth for energy deposition [50], which is the case in our study.

## Results and Discussion

### Raman spectra of irradiated MWCNT

**Figure 1:**
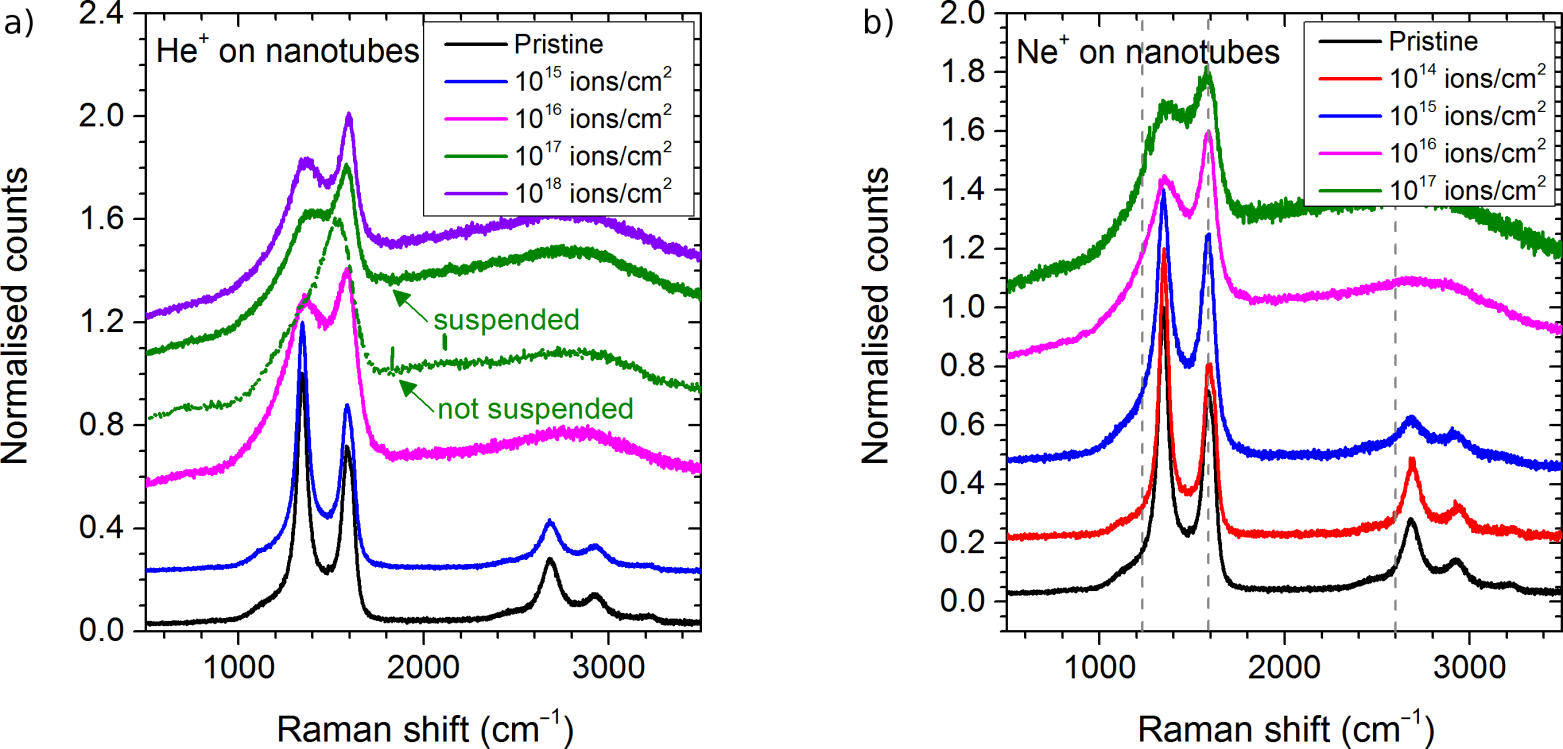
Raman spectra of multiwalled carbon nanotubes after irradiation with different fluences of a) 25 keV He^+^, and b) 25 keV Ne^+^ ions. A laser with a wavelength of 532 nm was used. All the spectra (except the one pointed out) were obtained on suspended free-standing MWCNTs.

Raman spectra have been recorded after irradiating the samples with different fluences of He^+^ and Ne^+^. The ion energy is always 25 keV. Figure 1 shows a representative spectrum for each fluence; a comparison of Raman spectra recorded in different locations of a same area with a same fluence can be found in Supporting Information File 1.

For the pristine samples two major peaks are visible: the D band at 1345 cm^−1^ and the G band at 1585 cm^−1^, the latter overlapping with the D’ band at 1615 cm^−1^. Some examples for deconvoluted spectra are shown for He^+^ irradiation in Supporting Information File 1. The D’ band is due to damage which is already present in the initial sample. Raman spectra of pristine samples similar to ours were observed by Lehtinen et al. for so-called bamboo MWCNTs [21], and by Ni et al. [22] and Nichols et al. [51] for CVD-grown MWCNTs. Compared to other data in literature the peak related to the D band is more intense than the one for the G band [1]. This indicates that quite a large amount of disorder is present in our initial samples.

For He^+^ irradiation at 10^17^ ions/cm^2^ two different spectra have been obtained. The spectra with D and G band has been obtained on suspended nanotubes while the spectra with only a broad G band has been recorded on a part of the sample where the nanotubes were lying directly on the Au grid. The appearance of one broad band indicates that the presence of the gold grid directly underneath the MWCNTs leads to increased damage formation in the sample.

**Figure 2:**
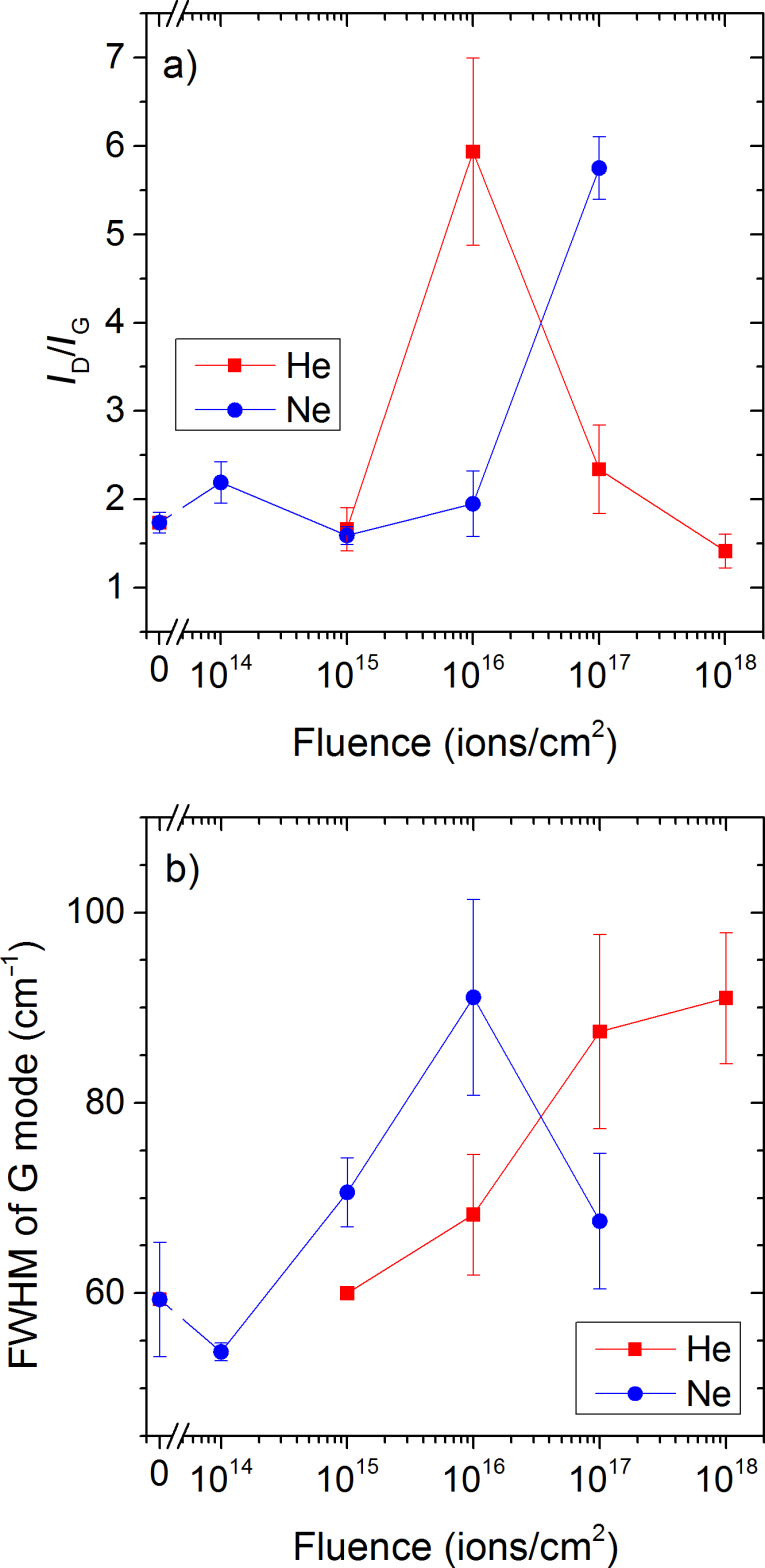
a) Ratio of intensities of D to G band as a function of fluence for 25 keV He and Ne irradiation, and b) full width at half maximum of the G band peak as a function of fluence for 25 keV He and Ne irradiation.

As a first indicator of the radiation-induced damage, we follow the evolution of the ratio of D band intensity to G band intensity *I*_D_/*I*_G_ with fluence (Figure 2a). For Ne^+^ irradiation, the ratio is increasing from low to high fluences. For He^+^ irradiation, *I*_D_/*I*_G_ increases up to a fluence of 10^16^ ions/cm^2^ to decrease afterwards to values which are close to those of the pristine sample. According to Aitkaliyeva and Shao the change in the *I*_D_/*I*_G_ trend can be related to structural changes in the following way: at lower fluences damage will be created without reducing significantly the number of sixfold rings. For higher fluences, in this work above 10^16^ ions/cm^2^, the number of sixfold rings starts to get reduced significantly, leading to an amorphisation of the sample [20]. For Ne^+^, *I*_D_/*I*_G_ increases to higher values than for He^+^ without getting to the point where the trend changes. The explanation can be related to the mass of the primary ion species. For Ne^+^, sputter yields are much higher than for He^+^, so that the damaged areas can be partially sputtered away, both from top and bottom sides of the sample.

An increase in the *I*_D_/*I*_G_ ratio followed by a decrease at higher fluences has also been observed for 140 keV He^+^ irradiation by Aitkaliyeva et al. [20] and for 60 MeV Ni^+^ and 120 MeV Au^+^ irradiation up to fluences of 10^14^ ions/cm^2^ by Jeet et al. [52]. For the latter, the maximum in *I*_D_/*I*_G_ is observed at much lower fluences (e.g., at 10^13^ ions/cm^2^ where collision cascades start to overlap) due to the higher damage formation in the MeV energy range. These results would probably also be valid for noble gas ion species, but Olejniczak et al. used only much lower fluences in their work for MeV irradiation and stayed most likely below the threshold for severe damage formation [53]. Niwase et al. carried out 25 keV He^+^ irradiation of graphite up to fluences of 10^18^ ions/cm^2^ and observed also a maximum in *I*_D_/*I*_G_ for temperatures up to 473 K [54]. Hence our data for He^+^ agrees well with results in literature. For Ne^+^ in this work, it is probable that the maximum in *I*_D_/*I*_G_ would occur at a higher fluence. The sample thickness may also have an influence on damage formation (cf. Simulation of He and Ne irradiation).

A second parameter giving information on damage formation is the evolution of the full width at half maximum (FWHM) of the G band peak with fluence (Figure 2b) [20]. For both He^+^ and Ne^+^ irradiation, the FWHMs follow similar evolutions at low fluences. For Ne^+^ irradiation, a maximum is observed at a fluence of 10^16^ ions/cm^2^, while it continues increasing until 10^18^ ions for He^+^ irradiation. For 140 keV and 3 MeV He^+^ irradiation, the FWHM of the G mode increases continuously for 140 keV and is almost constant for the higher energy [20,55]. The difference to our work (e.g., Ne^+^ irradiation) could be due to the higher impact energy of 140 keV. In addition, they did not consider the presence of the D’ band (cf. Supporting Information File 1). For 25 keV He^+^ irradiation of graphite by Niwase et al., a change in trend could be observed for fluences between 10^16^ and 10^17^ ions/cm^2^, with higher sample temperature pushing the maximum to a higher fluence [54]. Hence, their data at room temperature agrees well with ours.

The peak at 2700 cm^−1^ in the pristine sample, called G’, is an overtone of the D band and is an additional indicator for damage formation. It is caused by two-phonon scattering processes around the K point of the Brillouin zone and, like the D band, is sensitive to the defect density [1]. It gets significantly broadened with increasing fluence, indicating that the defect density is increasing, but significant difference between He^+^ and Ne^+^ irradiation is not observed. A decrease of the G’ band intensity is also observed for 140 keV He^+^ irradiation, but not for 3 MeV H^+^ irradiation up to a fluence of 5 × 10^5^ ions/cm^2^. Hence, low to high energy He irradiation causes more damage than MeV H^+^ ion bombardment [55].

### TEM observations

**Figure 3:**
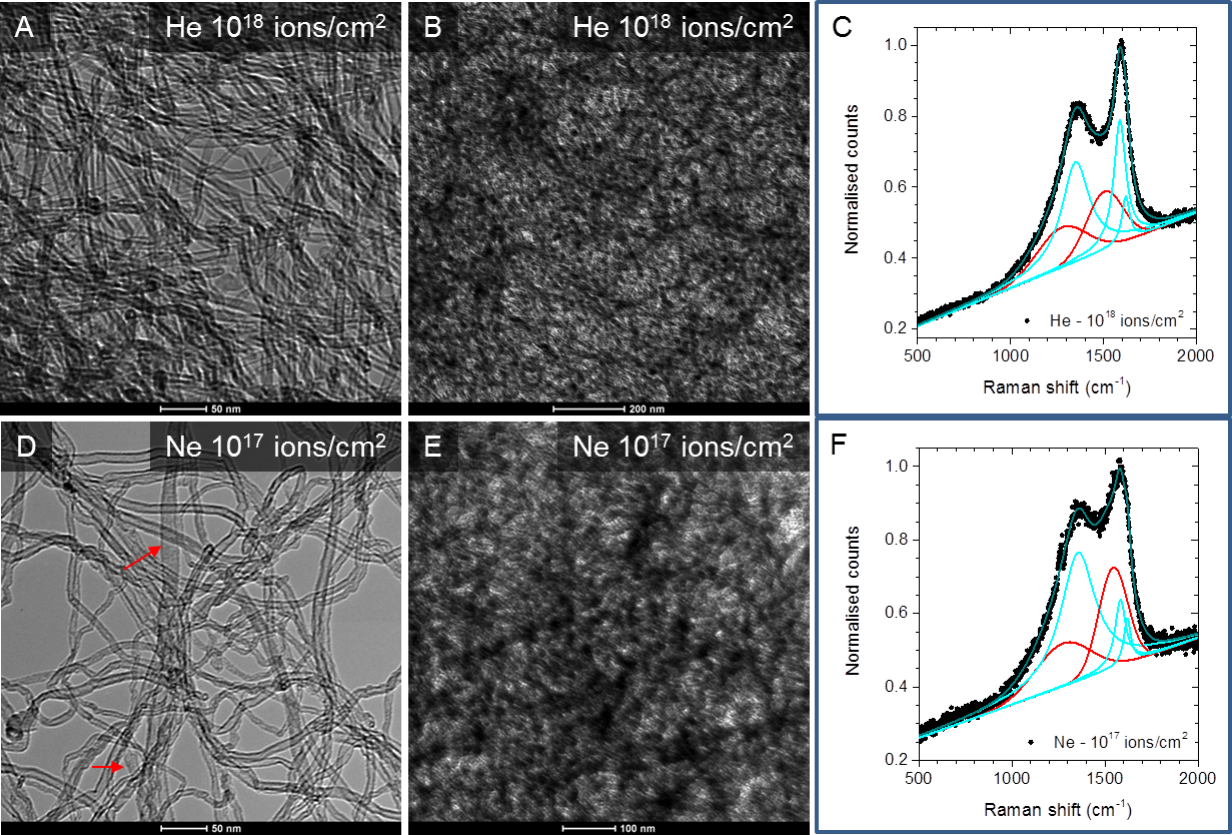
TEM images and Raman spectra after: (A–C) 25 keV He^+^ irradiation with a fluence of 10^18^ ions/cm^2^ (D–F) 25 keV Ne^+^ irradiation with a fluence of 10^17^ ions/cm^2^. While the thinner areas in both cases (A, D) appear to be relatively less damaged due to ion irradiation, the thicker sample areas (B, E) display diffuse contrast characteristic of partial amorphization, with most damage evident for the Ne^+^ irradiation. The arrows in (D) point to larger structures formed after ion irradiation. Plausible explanations are either by unzipping or other mechanisms of damage formation of MWCNT due to Ne^+^ irradiation. The structural features observed in these TEM images correlate very well with corresponding Raman spectra (C, F). In the deconvoluted spectra, Lorentz functions in cyan denote the D, G and D’ peaks while the Gaussian contributions from highly disordered areas are coloured in red.

The BF-TEM images of the ion-irradiated samples and corresponding Raman spectra are shown in Figure 3. The images were acquired after He^+^ irradiation with a fluence of 10^18^ ions/cm^2^ (Figure 3A,B) and after Ne^+^ irradiation with a dose of 10^17^ ions/cm^2^ (Figure 3D,E). For both He^+^ and Ne^+^ irradiation, thin areas (Figure 3A,D) show that the structure of MWCNT is preserved indicating that the structural damage is generally low. Occasionally, in Ne^+^ irradiation of thin areas, some larger structures (as indicated by arrows in Figure 3D) are observed suggesting possible local unzipping or damaging of MWCNT. Cross-linking between different MWCNTs can also be expected, since it has been observed in MD simulations of 32 keV Ar^+^ irradiation of SWNCNTs, but higher resolution would have been required in the regions with a thicker MWCNT layer [8].

**Figure 4:**
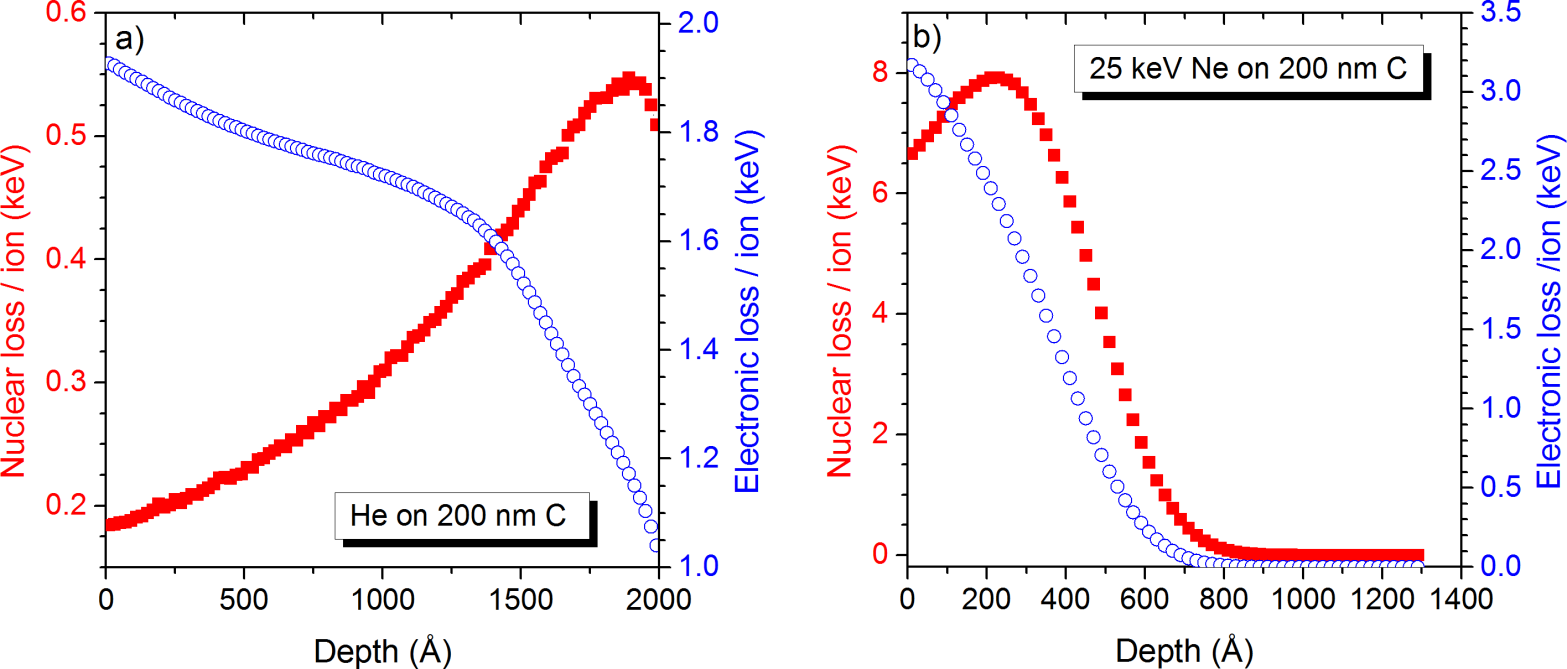
Nuclear and electronic energy loss as a function of sample thickness for a) He^+^ and b) Ne^+^ irradiation of suspended carbon.

Overall, the extent of structural damage was found to be thickness dependent (also cf. Supporting Information File 1). For both He^+^ and Ne^+^ irradiation, the thicker areas apparently suffered more damage than the thinner regions. For thinner samples, He^+^ irradiation with a fluence of 10^18^ ions/cm^2^ was found to have caused only a moderate amount of damage with the tubular structure of the MWCNT relatively preserved as shown in Figure 3B. In comparison Ne^+^ irradiation, even with a fluence which is lower by an order of magnitude than the He^+^, caused a larger amount of damage as evidenced by large areas of diffuse contrast indicating extensive damage and amorphization as shown in Figure 3E. The differences in the extent of damage observed by TEM in Figure 3B,E is consistent with the ratio of intensities of D to G band shown in Figure 2b. This agrees also with the simulation results discussed later which indicate a higher sputtering yield and a higher displacement due to Ne^+^ irradiation in comparison to He^+^ irradiation. The explanation for the thickness dependent damage is linked to the ion–solid interaction volume which for thicker samples is large enough for the collision cascade to fully develop and to deposit a much larger amount of energy in the sample thereby cause large structural damage. Overall, the structural information from TEM images and the chemical signal from Raman spectra are both consistent with each other and also with the simulations results shown below in Figure 4.

### Simulation of He and Ne irradiation

Numerical simulations have been carried out using the SDTRIMSP code [47]. As this code does not allow for the definition of tubes, the latter were simulated by carbon slabs of different thicknesses to represent MWCNTs overlapping. The experimental diameter of a MWCNT is about 10 nm which is the minimum thickness simulated in this work. The impact energy is equal to 25 keV, which is identical to experimental conditions. In this specific study the thickness of the carbon nanotube film, has a significant influence on the particle–sample interactions. For He irradiation the nuclear energy loss, which is mainly responsible for defect creation and sputtering, increases with depth up to a value of 190 nm. For samples with a thickness in between 30 and 200 nm, this means that more nuclear energy is deposited at the bottom of the sample than at its top (Figure 4). For Ne irradiation, the nuclear energy loss increases only up to a depth of 23 nm and decreases rapidly for larger depths. Hence, even for a single multiwalled carbon nanotube, the nuclear energy deposited at the bottom is not much higher than at its top. The graphs in Figure 4 have been obtained for the pristine sample. The aforementioned distributions of energy loss have an impact on the sputter yields at the top and bottom sides of the sample.

**Figure 5:**
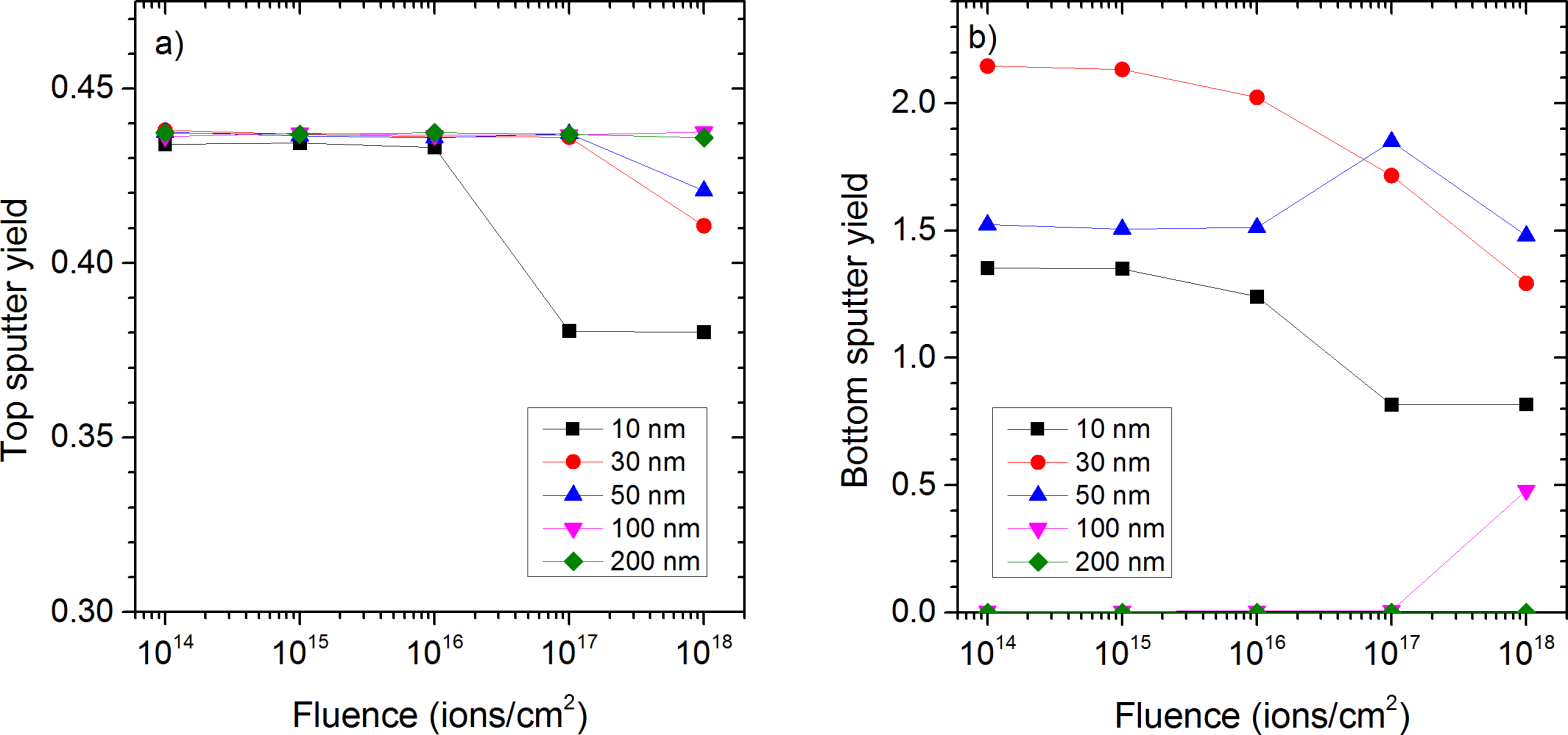
Sputter yield a) at the top, and b) at the bottom of the sample as a function of fluence for Ne irradiation of carbon samples of different thicknesses of 10–200 nm.

Depending on the film thickness, the collision cascade cannot fully develop which lowers the sputtering yield, or extends to the bottom of the sample causing also sputtering at that location. For sputtering from the top side of the sample, the influence of the film thickness is mainly seen for higher fluences of Ne irradiation (Figure 5a). For low fluences, sputter yields are all equal. At higher fluences, the sputtering yield of the 10 nm sample starts to decrease at first because the sample starts to be sputtered away and only a relatively small amount of the sample remains, leading to an increased transmission of the Ne ions and to a reduced amount of energy being deposited into the sample. The same behaviour occurs for the 30 nm sample at a higher fluence. For sample thicknesses above 50 nm, sputter yields remain constant as the sample is not thinned down sufficiently to influence the formation of the collision cascades.

For He irradiation, the sample thickness has no significant influence on the sputter yield on the top side of the sample. In any situation most of the energy is deposited deeply into the sample and only a small amount close to the sample surface producing some sputtering. At the fluence of 10^18^ ions/cm^2^, the sputter yield for He irradiation changes only from 1.4 × 10^−2^ for the 10 nm sample to 1.5 × 10^−2^ for the 200 nm sample (cf. Supporting Information File 1).

For sputtering from the bottom of the sample, the influence of the sample thickness is even more pronounced than for top side sputtering (Figure 5b). For Ne irradiation, the sputtering yield for the 10 nm sample starts at low fluences at a value of 1.35, which is more than three times higher than the top side sputtering yield. Once the samples start to get thinner, the bottom sputter yield lowers. The sputter yield being higher at the bottom than at the top can be explained by more energy being deposited at a depth of 10 nm than at the sample surface. This becomes even more pronounced for the 30 nm sample where the bottom sputter yield of 2.15 is about 5 times higher than the top side sputter yield. A similar ratio between sputter yields from sample top and bottom has been found for Hobler at al. for 20 keV Ar irradiation of amorphous Si membrane when sputtering from the top surface is maximum [50]. They showed that this maximum corresponds to a sample thickness which is equal to the mean depth for energy deposition. For the 50 nm sample, the optimum amount of energy for highest sputter yield is only deposited at the bottom side after thinning the sample down, which explains the maximum value at a fluence of 10^17^ ions/cm^2^. For the 200 nm sample, the film is too thick for a significant amount of energy being deposited at the backside of the sample. This explains the much lower sputter yields. The much higher sputter yields for Ne^+^ than for He^+^ could also explain why no in change in trend of *I*_D_/*I*_G_ is observed in the Raman spectra. For MWCNTs layers of a thickness of 1 to few tubes, the sputter rate could be higher or comparable to the damage accumulation rate, thereby avoiding large damage leading to a reduction of sixfold rings in the MWCNTs.

For He irradiation, the sputter yield at the bottom surface of the sample depends far less on fluence than for Ne irradiation due to the low sputter rate. However, there is a significant dependence on the sample thickness. It increases from 3.3 × 10^−2^ for the 10 nm to 4.3 × 10^−2^ for the 30 nm sample up to 1.3 × 10^−1^ for the 200 nm sample (cf. Supporting Information File 1). Hence, they are comparable to the Ne sputter yields at low fluence, i.e., before damage accumulates and the sample starts to be sputtered. For the sputtering from the top under He^+^ irradiation, the layer thickness has only an influence up to a value of about 50 nm, which means that only the top 50 nm of the cascade contribute to the sputtering from the top.

In the simulations, a homogenous film of carbon is considered. In reality, the carbon nanotubes form a 3D network which goes along with space in between the nanotubes and many top and bottom sides in the film. Hence, in the experiment the He and Ne ions spend some time in vacuum in between the MWCNTs, which leads to longer primary ion ranges than predicted by simulations. We consider that this will have no major effect on the comparison of simulation and experimental results except that a similar behaviour will be observed for a somewhat increased sample thickness in experiments. Hence, nanotubes at the sample bottom should be damaged and sputtered faster than those at the top side for samples with a thickness of a few tens of nm. This is also consistent with the TEM observation where the structure of MWCNTs preserved for the thin samples and damage is far more present in the thicker ones. The noble gas ion irradiation does not only cause sputtering but also the displacement of ions in the film. For the nanotube samples, they will cause damage and may lead to graphitised areas which regroup several or many nanotubes.

**Figure 6:**
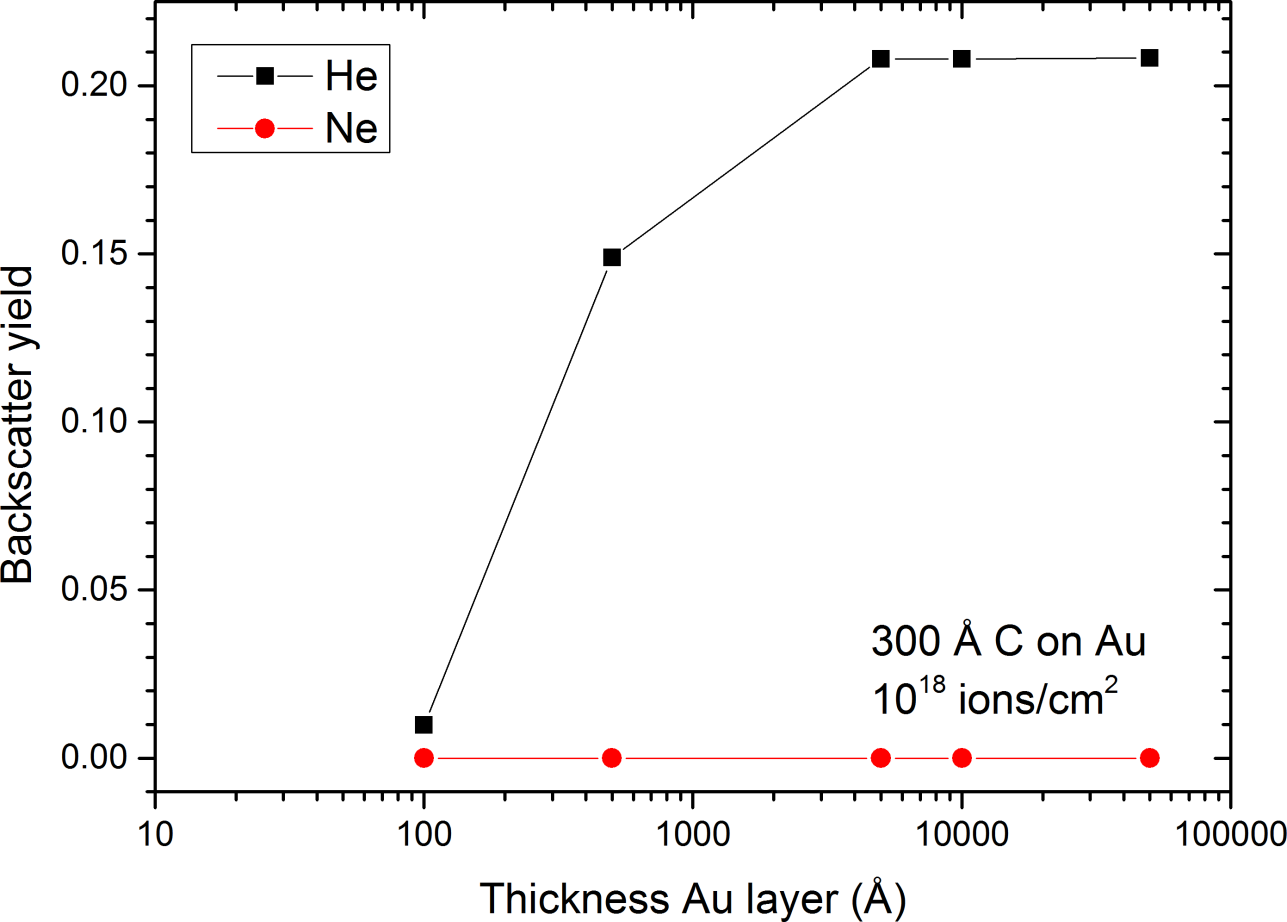
Backscatter yield as a function of gold thickness for He and Ne irradiation of a 30 nm carbon film deposited onto a gold grid. Results have been obtained by SDTRIMSP.

Besides the influence of the sample thickness, the substrate has also a significant impact on the irradiation induced damage in the MWCNTs. Although the Au grid has a fix thickness of 50 nm for the membrane and of several µm for the grid in between, simulations where carried out for a whole range of thicknesses from 10 nm to 5 µm to get a good understanding of how substrate thickness influences the processes at the MWCNT–Au interfaces. The Raman spectrum for the fluence of 10^17^ ions/cm^2^ with He^+^ ions shows that there is a difference between a suspended MWCNT film and a film deposited on a gold substrate, the nanotubes on top of the gold grid experiencing much more damage than the suspended ones (Figure 1). Backscattering of the noble gas species at the carbon–gold interface is here the main factor of importance. For the suspended films, the backscatter yields are very low with values below 2.6 × 10^−3^ for He and below 2.8 × 10^−5^ for Ne irradiation and the film thickness has only a minor influence (Table 1). For carbon films deposited on gold, the backscattering is largely enhanced for He irradiation, while the difference is only minor for Ne (Figure 6). The results are discussed for a 30 nm carbon film on a gold layer with varying thickness. For a 10 nm gold film, the He backscatter yield is already increased to 10^−2^ and reaches a plateau of 2.1 × 10^−1^ for gold films of 500 nm or thicker. The reason for the change in backscatter yield with the thickness of the Au substrate is the range of He ions. For the thin Au films of 10 and 50 nm, the collision cascade cannot fully develop with some part of the He ions being transmitted (about 1%), which leads to a lower backscatter yield than for substrate thicknesses equal to or above 500 nm.

**Table 1:** Backscatter yield averaged over a fluence of 10^18^ ions/cm^2^ for carbon film irradiation by 25 keV He^+^ and Ne^+^ irradiation.

Thickness of carbon film (nm)	Backscatter yield – He	Backscatter yield – Ne

10	3.0 × 10^−4^	1.1 × 10^−5^
30	1.2 × 10^−3^	1.9 × 10^−5^
100	2.6 × 10^−3^	2.8 × 10^−5^

**Figure 7:**
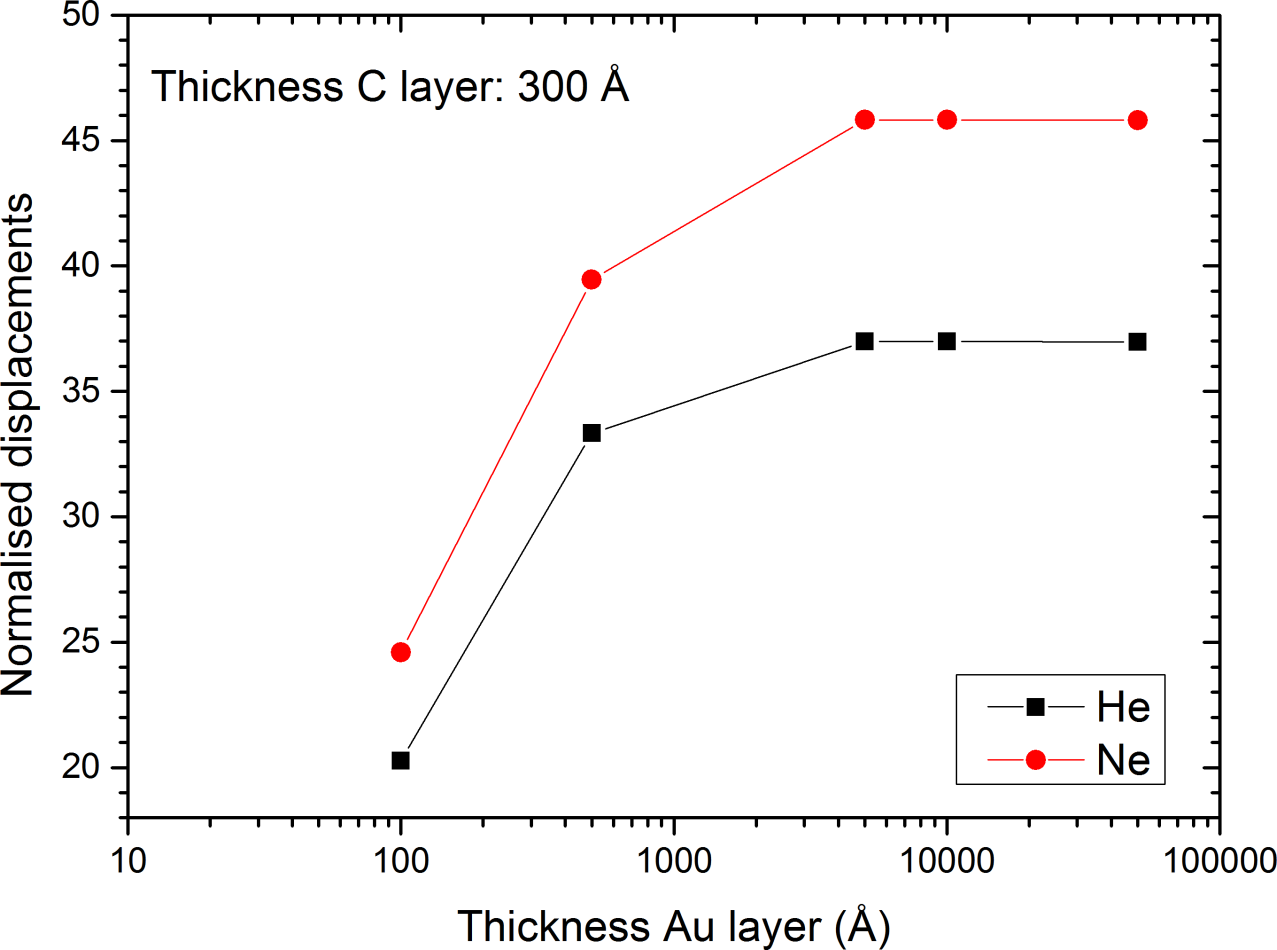
Displacements into the carbon layer normalised to incident ion as a function of gold thickness for He and Ne irradiation of a 30 nm carbon film deposited onto a gold grid. Results were obtained by SDTRIMSP.

The Au substrate does not only increase the backscatter yields by up to 3 orders of magnitude, but also the sputter events at the top side of the carbon sample. For the 30 nm carbon layer, the top-side sputter yield for He bombardment is equal to 1.5 × 10^−2^. This value increases slightly to 1.6 × 10^−2^ when the 30 nm carbon layer is put on a 10 nm gold film and to 5.2 × 10^−2^ for 30 nm carbon on gold films of 500 nm or thicker (Figure 7). This behaviour is equal to the change in the number of displacements in the carbon film normalised to the number of incident ions, where a plateau with maximum displacements is reached for gold layers of 500 nm or thicker. This means that only the first 500 nm of gold have an influence on the backscattering on the He in Au and the formation of the collision cascade in the above-lying carbon film. For Ne irradiation, the sputter yield at the top side is slightly reduced from 0.4 for carbon film to 0.3 for carbon–gold system. One possible reason is the development of the collision cascade at the carbon–gold interface and in the gold layer. Although the number of displacements in the carbon film normalised to the number of incident ions increases significantly with the thickness of the gold film, this increase does not contribute to a more efficient sputtering at the sample surface, i.e., they are occurring too far from the sample surface. The behaviour of both primary ion species is identical and similar to the backscatter yield of He. The number of normalised displacements increases with the thickness of the gold film to reach a plateau for a thickness of 500 nm. This shows that the presence of the gold changes the development of the collision cascades for He and Ne and leads to an increased formation of damage in the carbon film, which becomes visible in the Raman spectra as pointed out in Figure 1a. These results are also in agreement with the work of Krasheninnikov et al. where they showed for Ar irradiation of SWCNTs on Pt and SWCNTs on graphite that defect production is more important on substrates with heavy atoms than on those with light ones [56].

## Conclusion

In this work we used a correlative approach combining Raman spectroscopy with TEM and numerical simulations to study defect formation in suspended MWCNTs for 25 keV He^+^ and Ne^+^ ion irradiation at normal incidence for fluences ranging in between 10^14^ to 10^18^ ions/cm^2^. A new methodology based on Au TEM grids was developed and validated in order to ensure compatibility across the different experimental techniques. The influence of sample thickness on damage accumulation and amorphisation of the MWCNTs has also been discussed. For He^+^ irradiation, Raman spectra show that the sample starts to be heavily damaged starting from a fluence of 10^16^ ions/cm^2^. Nevertheless, the structure of the single CNTs is still visible for higher fluences on the TEM images. For Ne^+^ irradiation, no change in the *I*_D_/*I*_G_ trend is observed, indicating that damage accumulated without reducing significantly the number of sixfold rings. In TEM images, areas with thicker layers contain some spots where the initial CNT structure is no longer visible, but in most areas the tubular structure is maintained. This can be attributed to the sputter yields which are much higher for Ne^+^ irradiation than for He^+^ irradiation. Specifically, for the heavier species the sputter rates are high on the top and bottom side of the MWCNT layer, leading to the removal of matter on both sides of the sample. Due to the reduced thickness of the sample the damage caused by ion irradiation gets sputtered away, something which would not be observed in the bulk for implanted ions, and which could explain that no net reduction of sixfold rings can be observed up to a fluence of 10^17^ ions/cm^2^ for Ne^+^ irradiation. In future work, it would be interesting to investigate how ion irradiation with lower impact energies and different incidence angles would affect defect formation in the different tubes of a single MWCNT and between different MWCNTs and the evolution of CNT structure for various experimental conditions on the HIM.

## Experimental

### Sample preparation

Research grade Multi-Walled CNT (NC3100^TM^) samples were obtained from Nanocyl SA (Belgium). The MWCNTs have a diameter of about 10 nm. For analysing the samples before and after ion irradiation by Raman spectroscopy and TEM, the samples need to be prepared directly on TEM grids. Unfortunately, conventional TEM grids have an amorphous carbon membrane support film which is Raman active and therefore interferes with the Raman signal of the CNT. Hence, for this investigation, special Raman-inactive TEM grids were necessary. To fulfil this requirement, we obtained newly developed special TEM grids (300 mesh, R1.2/1.3) made of holey gold membrane (thickness 50 nm) UltrAuFoil^®^ from Quantifoil Micro Tools GmbH (Germany) [57].

A small quantity of the CNT sample was mixed with ca. 10 mL of ethanol and dispersed gently for a few minutes in a sonicator to obtain a well-dispersed suspension of the CNT. Thereafter, a few drops of the solution was deposited on the UltrAuFoil^®^ TEM grids and the excess liquid was removed using a filter paper. The Raman spectra of the freshly prepared samples deposited on TEM grids were consistent with the typical Raman signature of pristine MWCNT. In this way, the sample preparation protocol was validated.

Based on the contrast of TEM images of the pristine sample and the possibility to see individual MWCNTs the thickness is approximated to about 5 MWCNTs, which would be equivalent to about 50 nm. For thicker samples, it would no longer be possible to see the tubular structure.

### He^+^ and Ne^+^ irradiation

**Figure 8:**
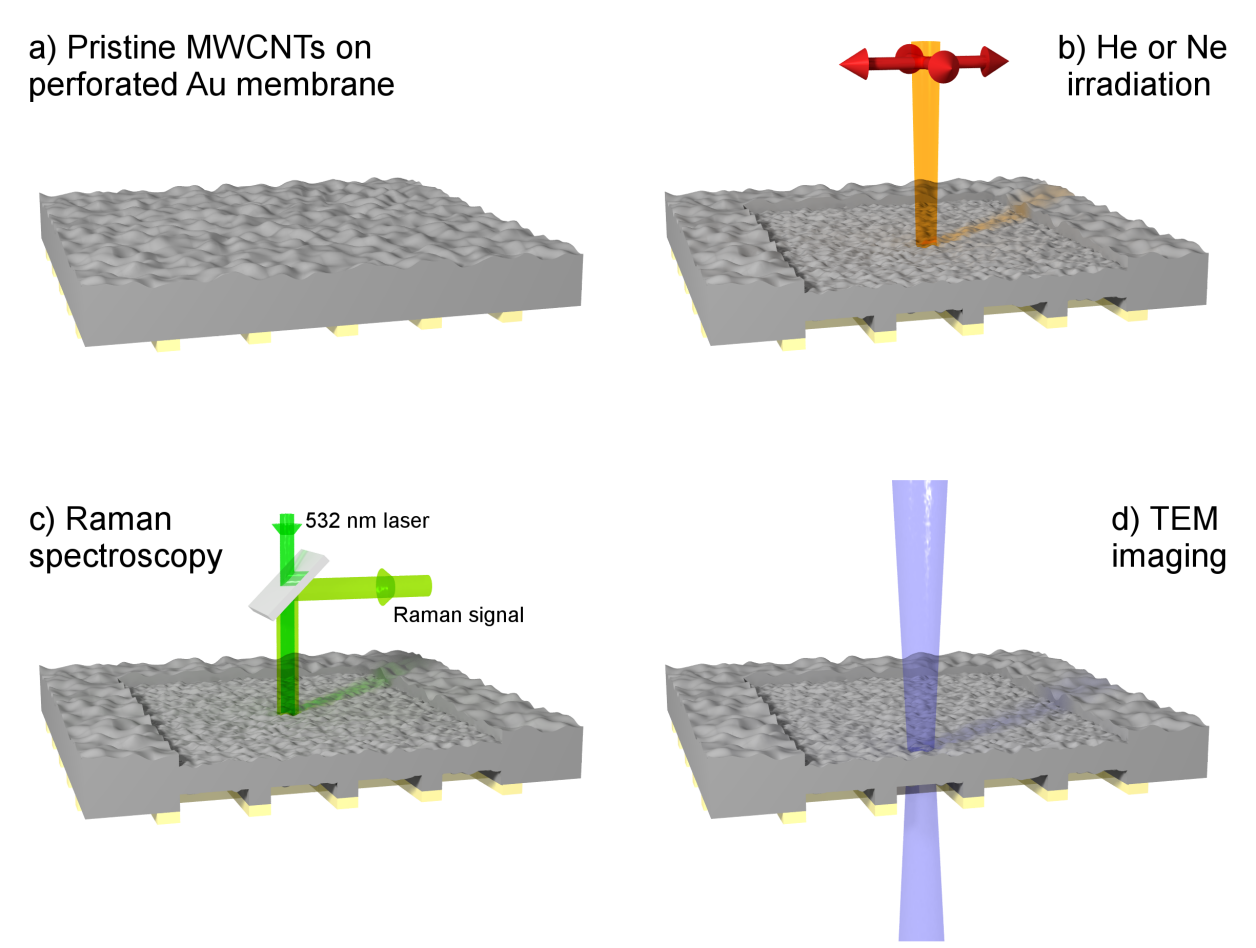
Schematic illustration of the sample configuration and the sequence of techniques used in this investigation: a) deposition of MWCNTs in a perforated Au TEM grid, b) irradiation with He^+^ or Ne^+^ ions with a fluence of 10^14^–10^18^ ions/cm^2^, c) Raman spectroscopy in the irradiated area with a 532 nm laser, and d) TEM analyses in the irradiated area.

The TEM grids with MWCNT samples were first mounted on a stub holder which takes up to 4 free-standing TEM grids (PLANO GmbH, Product G3662) and were irradiated with He^+^ and Ne^+^ ion beams in an Orion Nanofab Instrument (Zeiss) [33]. The He^+^ and Ne^+^ ions generated in the gas-field ion source (GFIS) column were accelerated to 25 keV with a primary current from 15 pA to 45 pA. The beam at normal incidence was raster scanned over a surface of 40 × 40 µm^2^ to 60 × 60 µm^2^ (Figure 8) for total irradiation fluences of the MWCNT samples ranged from 10^14^ to 10^18^ ions/cm^2^. Beam position and primary ion fluence were controlled by the Fibics Nanopatterning and Visualisation Engine (NPVE) (http://www.fibics.com/). The FIBICS nano-patterning software allows for high-performance milling and ion-beam etching and takes changes in ion current during long-time irradiation into account. The scan mode with spot overlap was used for ion beam positioning, leading to a homogeneous irradiation of the sample. The experimental conditions are given in Table 2.

**Table 2:** Experimental conditions for He^+^ and Ne^+^ irradiation on the HIM. Irradiation time is calculated based on primary ion current and changes of the latter during ion irradiation.

		Fluence (ions/cm^2^)				
		10^14^	10^15^	10^16^	10^17^	10^18^

He	Current (pA)	15	15	15	45	45
	Crater size (µm^2^)	60 × 60	60 × 60	60 × 60	40 × 40	40 × 40

Ne	Current (pA)	20	20	20	40	
	Crater size (µm^2^)	60 × 60	60 × 60	60 × 60	40 × 40	

### Raman spectra

Raman scattering measurements were performed with an inVia Renishaw Reflex Raman Microscope in micro-Raman mode, with an edge filter with spectral cutoff at 80 cm^−1^ and a 2400 grooves/mm grating for the analysis of the scattered light. A frequency doubled Nd:YAG laser (532 nm) was used as excitation source. The laser beam was focused through a microscope objective with magnification ×100L and numerical aperture 0.85, which results in a spot size of about 0.76 µm. Laser power was not higher than 0.44 mW. For each fluence, 2–4 measurements were done in different locations of every crater created by ion irradiation (Figure 8). For deconvolution of the Raman spectra, the method by Ribeiro-Soares et al. has been used [58]. The peaks for the D band at 1345 cm^−1^, the G band at 1585 cm^−1^ and the D’ band at 1615 cm^−1^ have been fitted by Lorentzian functions while Gaussian functions have been used to fit the contribution from highly disordered areas close to 1250 cm^−1^ and 1490 cm^−1^. In order for the fitting algorithm to converge, the peak positions of the Gaussian peak at 1250 cm^−1^ and the D’ peak needed to be fixed. After fitting, integrated peak intensities of the D and G bands and the full width at half maximum (FWHM) of the G and D’ bands have been extracted from every Raman spectra. Error bars have been created using the standard deviation of the different parameters.

### TEM analyses

The TEM used in this study was a FEI Tecnai G2 F20 operating at 120 kV. Note that this TEM was adapted previously to also enable in situ secondary ion mass spectrometry experiments as described elsewhere, which leads to a modified octagon and pole-pieces [59]. The bright-field (BF) TEM images were recorded in the He^+^ and Ne^+^ craters using Gatan on-axis CCD camera with 2k × 2k pixel array (Figure 8). Raman spectroscopy was done prior to TEM analysis so that the Raman signal is purely due to ion bombardment without any possible contribution from electron irradiation associated with TEM imaging.

### SDTRIMSP simulations

Simulations on He and Ne irradiation of the carbon nanotubes were carried out using the SDTRIMSP code [47] which is based on the simulation codes TRIM [60–61] and TRIDYN [62–63]. In addition to previous codes, SDTRIMSP includes the option to consider the outgassing of atoms in a sample [64]. This is required for the simulation of helium and neon ion bombardment of organic samples. The diffusion coefficients for the different noble gas species have been taken from previous work [43]. For He, a diffusion coefficient of 4.8 × 10^−6^ cm^2^ s^−1^ was used, and for Ne a value of 1.1 × 10^−6^ cm^2^ s^−1^. The irradiation was simulated for 25 keV ion impacts at normal incidence with a fluence of up to 10^18^ ions/cm^2^ which corresponds to the experimental conditions used on the helium ion microscope. During the simulations, the KrC potential has been used for interatomic interactions, the Oen–Robinson model for electronic stopping and the Gauss–Mehler method with 16 pivots for integration. The surface binding energy for the noble gas species is calculated using sbe_RG_ = *q*_RG_∙Es_RG_ + *q*_C_∙0.5∙(Es_RG_ + Es_C_) and the surface binding energy for carbon is calculated using sbe_C_ = *q*_RG_∙0.5∙(Es_RG_ + Es_C_) + *q*_C_∙Es_C_∙sbe_RG_ and sbe_C_ are the surface binding energy of noble gas and carbon atoms in the target, Es_RG_ and Es_C_ are the atomic surface binding energies for the noble gas species and carbon and *q*_RG_ and *q*_C_ are the atomic fractions [47].

In SDTRIMP only massive samples or layer can be used. Therefore different carbon films with a thickness of 10 to 200 nm have been created. To study the influence of the gold TEM grid on which the nanotubes were suspended, a two-layered sample with 30 or 100 nm thick carbon film on top of a 10 to 5000 nm thick gold layer have been prepared.

## Supporting Information

File 1 Additional information on deconvolution of Raman spectra, TEM imaging and numerical simulations.
