# Supplementary material for: Defect formation in multiwalled carbon nanotubes under low-energy He and Ne ion irradiation
Source: Beilstein J Nanotechnol. 2018 Jul 9;9:1951–63. doi: 10.3762/bjnano.9.186 (PMC6071685; doi:10.3762/bjnano.9.186)
Supplement: File 1 — Additional information on deconvolution of Raman spectra, TEM imaging and numerical simulations. [file Beilstein_J_Nanotechnol-09-1951-s001.pdf]

Supporting Information
for
Defect formation in multiwalled carbon nanotubes under low-energy He and Ne ion irradiation

Santhana Eswara[1], Jean-Nicolas Audinot[1], Brahime El Adib[2], Maël Guennou[2], Tom Wirtz[1] and Patrick Philipp[*1]

Address: [1]Advanced Instrumentation for Ion Nano-Analytics (AINA), MRT Department, Luxembourg Institute of Science and Technology (LIST), 41 rue du Brill, L-4422 Belvaux, Luxembourg and [2]Materials Research and Technology Department, Luxembourg Institute of Science and Technology, 41 rue du Brill, L-4422 Belvaux, Luxembourg

Email: Patrick Philipp - patrick.philipp@list.lu

* Corresponding author

Additional information on deconvolution of Raman spectra, TEM imaging and numerical simulations

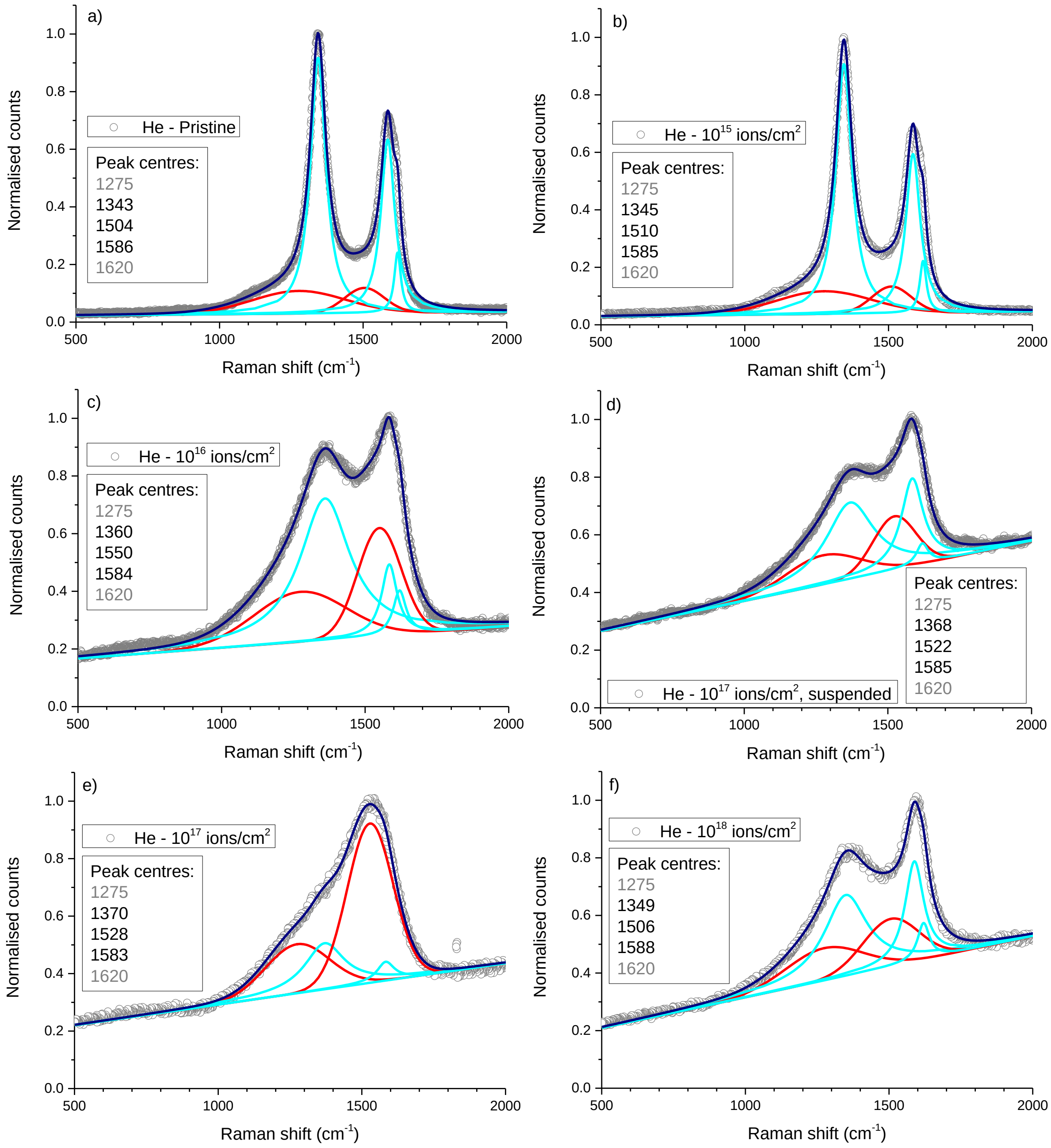

Figure S1: Deconvolution shown for a) the pristine sample and different fluences under He^+ irradiation: b) 10^{15} ions/cm^2, c) 10^{16} ions/cm^2, d) 10^{17} ions/cm^2 for suspended CNTs, e) 10^{17} ions/cm^2 for CNTs on Au substrate, f) 10^{18} ions/cm^2. Open circles show the experimental Raman spectra, the different lines the different contributions to the fit (in cyan the Lorentz functions for the Raman peaks, in red the Gaussian contribution from highly disordered areas) and the sum of them (ion blue). For e) the surface of the peak at 1620 cm^{-1} is equal to 0.

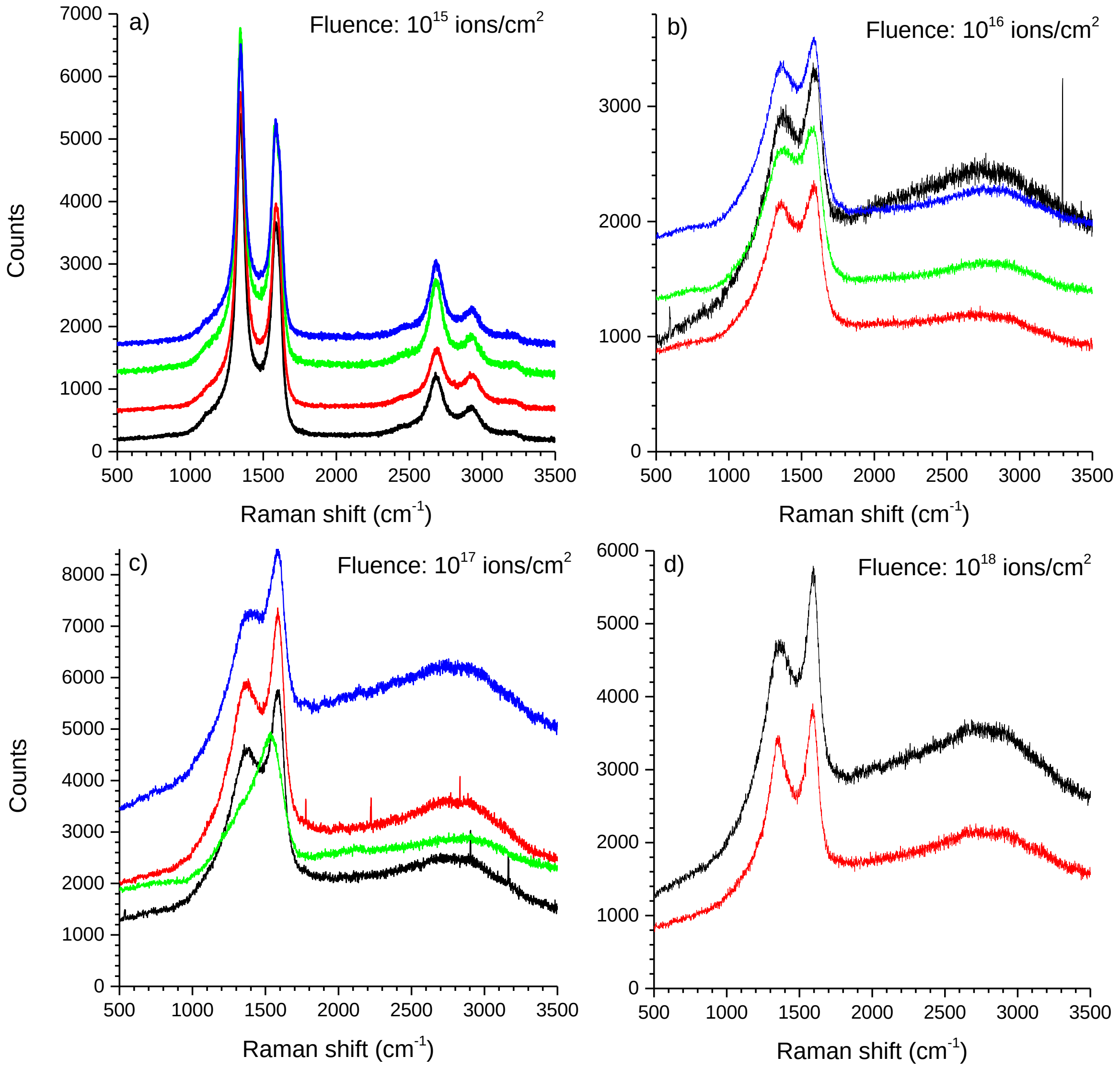

Figure S2: Raw Raman spectra shown for He^+ irradiation at different fluences: a) 10^{15} ions/cm2, b) 10^{16} ions/cm^2, c) 10^{17} ions/cm^2, d) 10^{18} ions/cm^2. For clearer visualization an offset along the y axis has been applied to the different spectra.

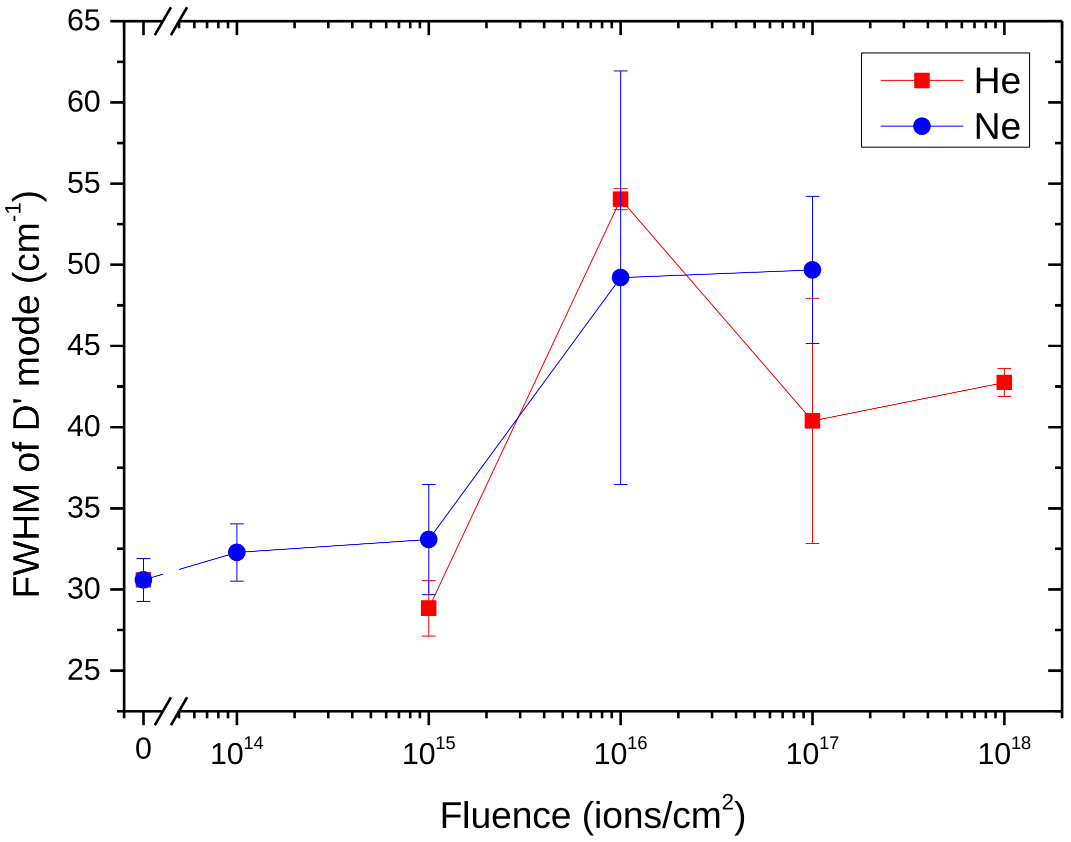

Figure S3: Evolution of the FWHM of the D' band with fluence for 25 keV He^+ and Ne^+ irradiation of MWCNTs. The data follows exactly the same trend than for the G band.

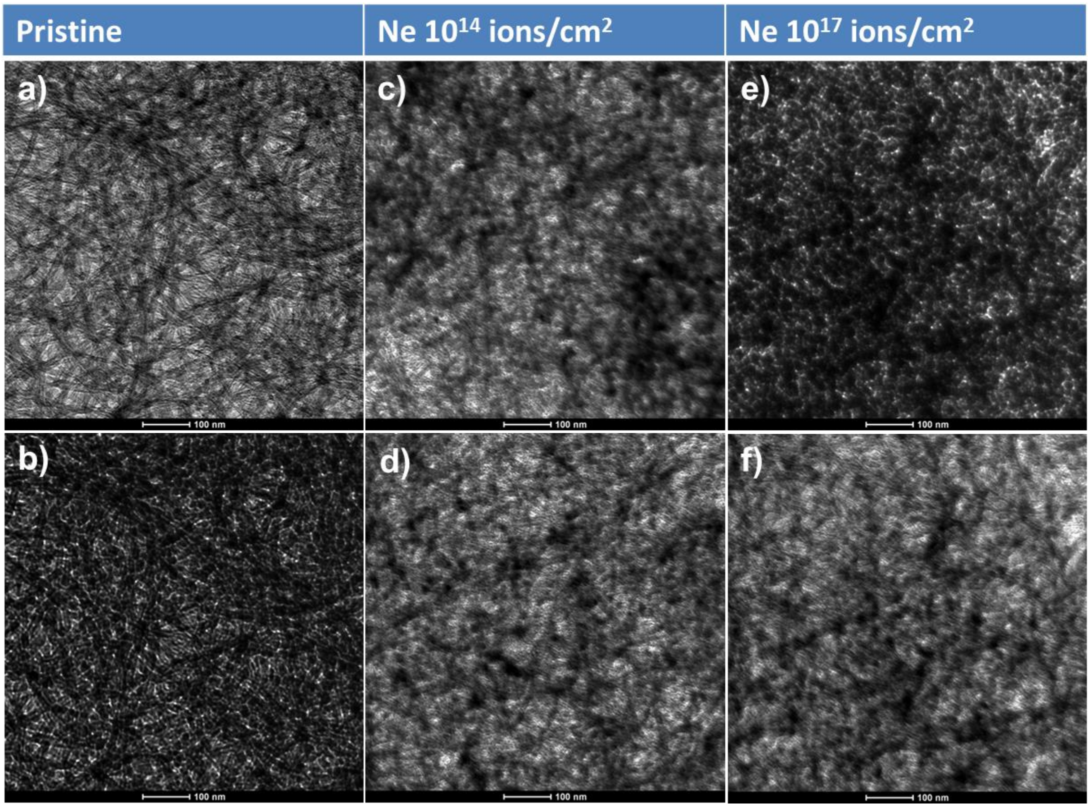

Figure S4: In order to assess the through-thickness variation in the extent of damage due to ion irradiation, TEM images were taken at different objective lens focus values. While the contrast changes uniformly for pristine samples (a,b), the images acquired after Ne^+ irradiation with fluences of 10^{14} (c, d) and 10^{17} (e, f) ions/cm^2 show strong and irregular variation of contrast indicating a clear through-thickness variation in the extent of damage. These results are consistent with the SRIM simulations which indicate that the top surface experience less damage than the bottom surface for thin samples, i.e. nuclear stopping is highest at a depth of 30 nm below the sample surface.

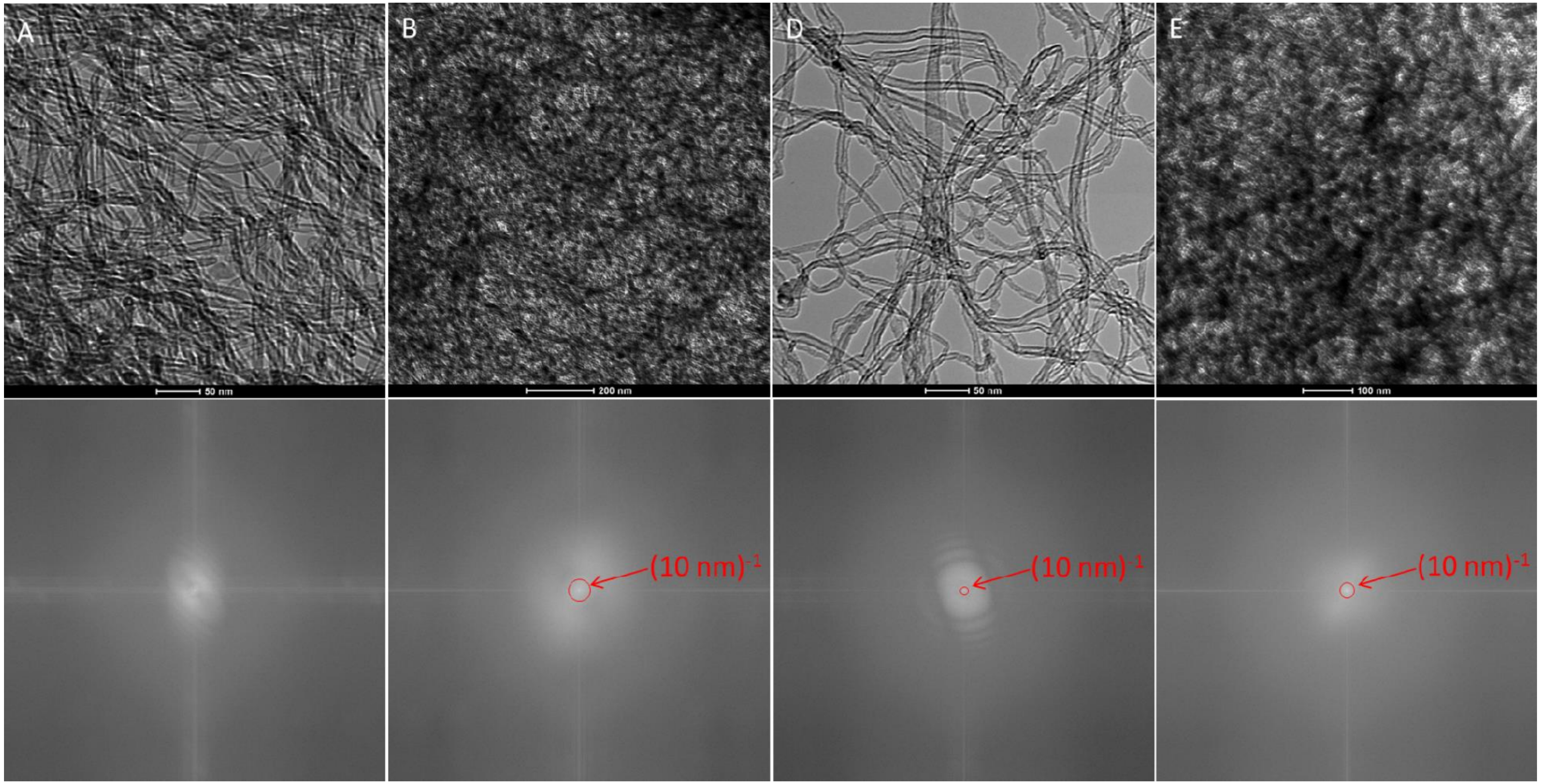

Figure S5: TEM images (same as in Fig. 3A, B, D, E respectively) and their Fast Fourier Transforms (FFT). The spatial frequency corresponding to the diameter of the MWCNTS (10 nm) are indicated by red circles in the FFTs. Note, as the FFT of the first image show slight objective lens astigmatism, the red circle is not drawn on it. Intensities present outside the red circles are attributed mainly to defective nanotubes and amorphized material, in consistence with Raman spectra.

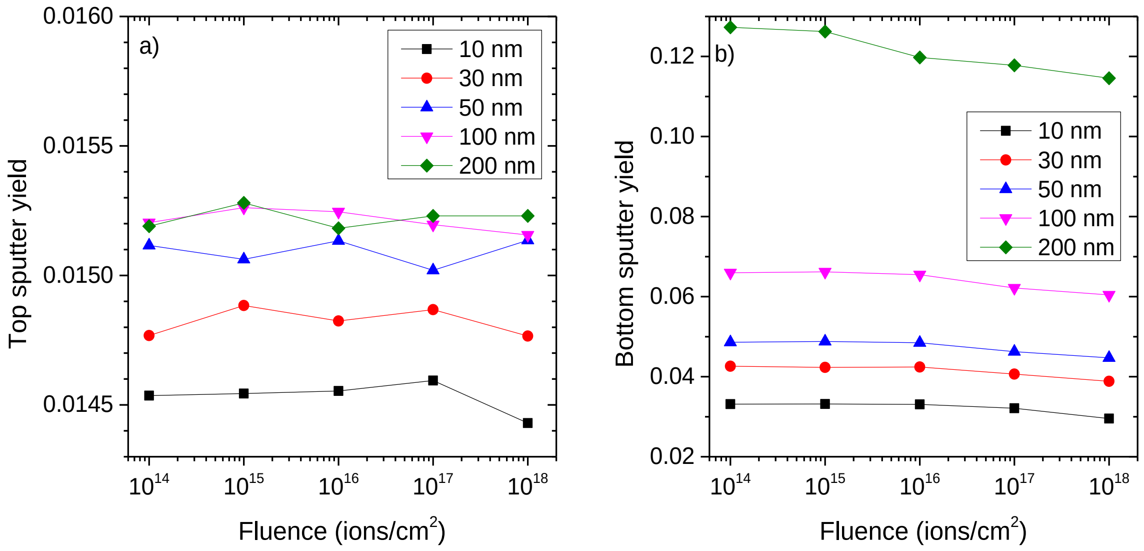

Figure S6: Sputter yields for the a) top and b) bottom side of the sample under He^+ irradiation. Due to the higher nuclear stopping at the bottom of the sample, bottom sputter yields are higher than top sputter yields. Due to the overall small sputter yields, their value does not change significantly with fluence.
